# Isotretinoin-Induced Distal Ileitis Mimicking Crohn’s Disease

**DOI:** 10.7759/cureus.33766

**Published:** 2023-01-14

**Authors:** Joana Pereira-Nunes, Gabriela Reis, Susana Teixeira, Nélia S Gaspar, Céu Espinheira, Eunice Trindade

**Affiliations:** 1 Department of Pediatrics, Centro Hospitalar Universitário de São João, Porto, PRT; 2 Department of Gynecology-Obstetrics and Pediatrics, Faculty of Medicine of Porto University, Porto, PRT; 3 Department of Pediatrics, Unidade Local de Saúde do Baixo Alentejo, Beja, PRT; 4 Department of Pediatrics, Centro Hospitalar de Trás-os-Montes e Alto Douro, Vila Real, PRT; 5 Department of Pediatrics, Centro Hospitalar Médio Tejo, Torres Novas, PRT; 6 Pediatric Gastroenterology Unit, Centro Hospitalar Universitário de São João, Porto, PRT

**Keywords:** isotretinoin, inflammatory bowel disease, ileitis, drug-induced bowel injury, crohn’s disease

## Abstract

Terminal ileitis is a common condition defined as inflammation of the terminal portion of the ileum, which is typically associated with inflammatory bowel disease (IBD), classically Crohn’s disease (CD). However, it can have other etiologies, including drug-induced ones. Isotretinoin is an effective and commonly used treatment for acne vulgaris, presenting multiple adverse effects. There have been discussions over its association with enteric inflammation, particularly over IBD emergence risk. We report a case of a previously healthy 17-year-old female who presented transitory clinical, laboratory, imaging, and endoscopic evidence of distal ileitis, temporally related to extended isotretinoin treatment and mimicking CD. Repeated clinical, laboratory, imaging, and endoscopic reassessment after isotretinoin discontinuation confirmed an almost complete resolution of the condition, avoiding IBD misdiagnosis and specific medication initiation. Our case highlights the differential diagnosis of ileitis as being of critical importance to avoid further unnecessary diagnostic investigations and inadequate treatment. Serial re-evaluation may be of key importance to reach a final diagnosis. Although recent literature suggests that isotretinoin is not associated with an increased IBD risk, our case highlights the possibility of it inducing small bowel injury and inflammation, similar to what has been reported with other drugs.

## Introduction

Terminal ileitis is a common condition defined as inflammation of the terminal portion of the ileum, comprising erosions, ulcers, aphthous ulcers, nodular, edematous and/or erythematous mucosa. [1]. The clinical presentation is usually characterized by acute abdominal pain, particularly located at the right lower quadrant, and/or diarrhea, with some cases exhibiting obstructive symptoms, hemorrhage, and/or extra-intestinal manifestations, depending on its etiology [2,3].

Terminal ileitis is frequently associated with inflammatory bowel disease (IBD), especially Crohn’s disease (CD). It has also been associated with backwash ileitis due to ulcerative colitis [2,4]. However, it may present multiple possible etiologies, such as infections, malignancies, vascular, autoimmune and/or inflammatory disorders [1-3,5,6]. Evidence of drug-related inflammation of the small bowel and the remaining gastrointestinal tract has also been described, with nonsteroidal anti-inflammatory drugs (NSAIDs) being a classic example [2,5-8].

Isotretinoin or 13-cis retinoic acid is a vitamin A-derived retinoid, frequently used for moderate to severe acne vulgaris treatment [9-11]. Overall, it is a very effective medication but presents multiple side effects [9-11]. There have been discussions over its association with enteric inflammation, particularly over IBD emergence risk [10,12,13].

We report a case of a previously healthy 17-year-old female who presented transitory clinical, laboratory, imaging, and endoscopic evidence of distal ileitis, temporally related to extended isotretinoin treatment, mimicking CD. Withdrawal of this medication resulted in significant improvement.

## Case presentation

A 17-year-old female was referred to our Pediatric Gastroenterology Unit due to suspicion of inflammatory bowel disease. She had normal growth, and her personal and family history were unremarkable except for a two-year history of six months periods of isotretinoin treatment because of acne. She was not taking any other medication.

She presented a four-month history of recurrent episodes of severe hypogastric abdominal pain, occasionally associated with vasovagal syncope due to the pain intensity. She had daily normal passing stools, but during these episodes, she presented watery and nonbloody diarrhea. She denied any constitutional symptoms, including fever, malaise, asthenia, loss of appetite, weight loss, nausea, vomiting or joint involvement. Owing to these clinical manifestations, an enhanced magnetic resonance enterography (MRE) was performed by her attending physician. It revealed a 7 cm extension of discontinuous concentric thickening of the last ileal loop (6 mm), presenting diffusion restriction and contrast hyperenhancement. The submucosa displayed slight increased T2 signal, indicative of edema, and there was engorgement of the mesenteric vessels that supplied it. These findings were considered compatible with inflammatory bowel disease, namely active CD.

**Figure 1:**
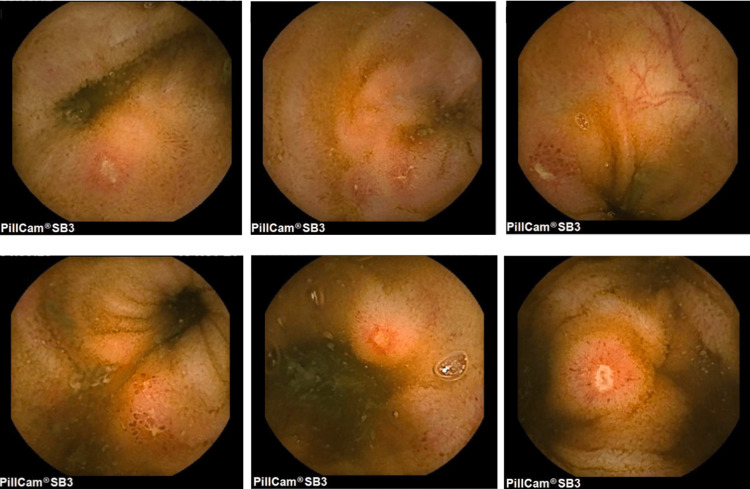
First video capsule endoscopy revealing multiple stellate distal ileum ulcers of variable sizes.

The laboratory workup performed did not display anemia or an acute-phase response, presenting normal C-reactive protein (CRP) and erythrocyte sedimentation rate (ESR). Serum immunoglobulins were in the normal range for age and celiac serologies were negative. Perinuclear antineutrophil cytoplasmic antibodies (P-ANCA) and anti-saccharomyces cerevisiae antibodies (ASCA) were also negative. Remarkably elevated fecal calprotectin level was present: 204 mcg/g. Additional laboratory investigations were non-contributory, including an infectious and inflammatory workup. Her esophagogastroduodenoscopy and complete ileocolonoscopy were normal. Random biopsies throughout the terminal ileum, colon and rectum were obtained, revealing a slight colic lymphoplasmacytic inflammatory infiltrate. Due to the association of the clinical manifestations, imaging evidence of terminal ileitis, elevated fecal calprotectin, and absence of typical endoscopic features of CD, a video capsule endoscopy (VCE) was scheduled to exclude small bowel disease. It revealed multiple stellate distal ileum ulcers of variable sizes (Figure 1). Areas of mucosal erosion were also visible but there were no signs of active bleeding.

**Figure 2:**
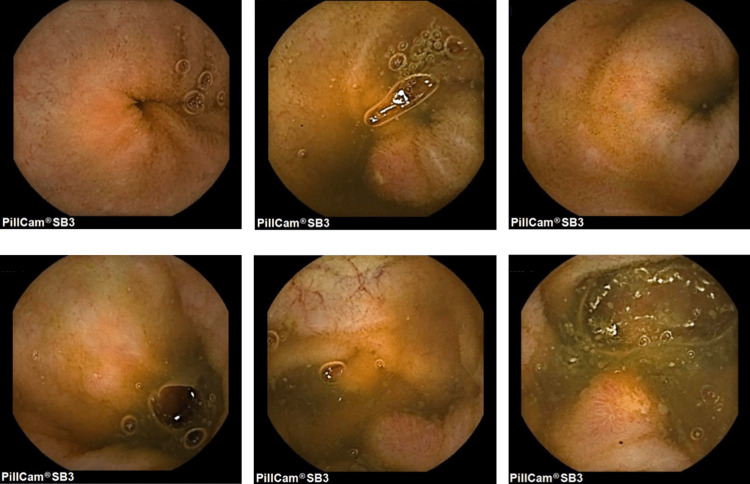
Second video capsule endoscopy displaying significant improvement, with identification of only two small ileal ulcers.

On her one-month clinical reassessment, the patient reported clinical improvement without any new episode of abdominal pain. She denied any life modifications apart from having self-discontinued isotretinoin treatment since her last appointment. Although a course of budesonide treatment was initially considered, a repeated assessment was performed. Repeated measurements of fecal calprotectin levels showed serial normal values (<30 mcg/g). An abdominal ultrasound confirmed the resolution of previous reported ileal thickening. The new VCE, performed three months later, displayed significant improvement with identification of only two small ileal ulcers (Figure 2).

Fourteen months later the patient remains asymptomatic, without new episodes of abdominal pain or diarrhea. Serial fecal calprotectin levels remain normal. Isotretinoin treatment was not resumed. Due to the history and time related improvement with isotretinoin discontinuation, an isotretinoin-induced distal ileitis was diagnosed.

## Discussion

Despite being a common condition, the accurate diagnosis of the multiple etiologies of terminal ileitis may represent a difficult clinical challenge. However, establishing its specific cause is of paramount importance because misdiagnosis may result in delays or errors in patient management [14]. We reported a case of a 17-year-old female who presented clinical manifestations, elevated fecal calprotectin and imaging, and VCE evidence of distal ileitis, which led to an unconfirmed IBD suspicion. The history and time-related improvement with isotretinoin discontinuation suggested that this transitory ileum inflammation was possibly related to its use. The clinical, laboratory, imaging and endoscopic reassessment after medication withdrawal allowed the confirmation of almost complete ileitis resolution. This allowed us to avoid the initiation of specific IBD therapy.

Isotretinoin was approved by the United States Food and Drug Administration (FDA) for acne treatment in 1982 [10]. Since then, it has been considered the most clinically effective anti-acne therapy [10]. However, in addition to teratogenicity, multiple other adverse effects have been associated with it [9,10,15]. Mucocutaneous, ocular, lipid and hepatic effects constitute some of the most reported ones [9,10,15]. During the last few years, there have been concerns about a possible association between isotretinoin and new-onset or worsening of IBD [10,12,13,16]. Although there still are conflicting data, recent data suggest that its use is not apparently associated with an increased risk of developing ulcerative colitis or CD [10,12,13,16].

Fecal calprotectin is a biomarker for intestinal inflammation since it is a useful tool to distinguish between organic and functional pathology [17,18]. Since it can predict mucosal healing and the response to treatments, fecal calprotectin is also commonly used in the follow-up of confirmed IBD patients, being a convenient non-invasive marker of inflammation in such patients [17-19]. Our patient presented an initial elevated fecal calprotectin (>100 mcg/g), namely > 200 mcg/g which is considered especially significant [17]. At the time of the presentation, our patient was not taking any other medications beyond isotretinoin, and additional investigations, including infectious, inflammatory and autoimmune workup were normal, excluding other possible explanations for the increased fecal calprotectin and reflected bowel inflammation. Also, perinuclear anti-neutrophil cytoplasmic antibody (p-ANCA) and anti-Saccharomyces cerevisiae antibody (ASCA) markers, with good specificity but poor sensitivity for IBD [20], were negative.

Our patient presented clinical, laboratory, and imaging evidence of small bowel inflammation, in particular of the terminal ileum, which improved after self-discontinuation of isotretinoin medication, without any specific or directed treatment. This was reflected by the combination of clinical and endoscopically ileum injury appearance improvement on VCE, as well as normalization of fecal calprotectin. Although our patient still presents a short follow-up time and further study in this area are required to establish a stronger correlation, symptoms correlated well with the timing of her isotretinoin use. When she self-discontinued the acne therapy, global evidence of small bowel inflammation improved, thus confirming a reasonable suspicion that her manifestations were possibly associated with it.

Although not associated with an increased IBD risk, our patient highlights the possibility of isotretinoin inducing small bowel injury, similar to what has been reported with other drugs [8]. As in our case, in patients using isotretinoin who are found to have evidence of intestinal inflammation, without any other obvious cause, it should be considered as a possible instigator. In these cases, isotretinoin discontinuation should be considered and clinical, analytical, imaging and endoscopic reassessment repeated, in order to avoid the establishment of an IBD misdiagnosis. Although further studies are warranted to obtain more data about possible isotretinoin-induced bowel injury, it might be reasonable to counsel patients on this potential isotretinoin adverse effect when starting this medication.

## Conclusions

Our case highlights ileitis as not always being a synonym for IBD. Differential diagnosis, although sometimes difficult, is of critical importance to avoid further unnecessary diagnostic workup and inappropriate treatment. Repeated clinical and diagnostic reassessment may be of key importance to reach a final diagnosis. Although not reported as being a causative IBD factor, we consider that, in the absence of other causes, isotretinoin is a potential trigger for transitory enteric inflammation and injury, particularly of the small bowel.
